# GET.ON Mood Enhancer: efficacy of Internet-based guided self-help compared to psychoeducation for depression: an investigator-blinded randomised controlled trial

**DOI:** 10.1186/1745-6215-15-39

**Published:** 2014-01-30

**Authors:** David Daniel Ebert, Dirk Lehr, Harald Baumeister, Leif Boß, Heleen Riper, Pim Cuijpers, Jo Annika Reins, Claudia Buntrock, Matthias Berking

**Affiliations:** 1 Leuphana University, Innovation Incubator, Division Health Trainings online, Rotenbleicher Weg 67, Lüneburg 21335, Germany; 2 Philipps University Marburg, Department of Psychology; Clinical Psychology and Psychotherapy, Marburg, Germany; 3 Department of Medical Psychology and Medical Sociology, Faculty of Medicine, University of Freiburg, Freiburg, Germany; 4 Department of Rehabilitation Psychology and Psychotherapy, Institute of Psychology, University of Freiburg, Freiburg, Germany; 5 GGZ inGeest, Regional Mental Health Service Centre, VU University Medical Centre, Amsterdam, The Netherlands; 6 Department of Clinical Psychology and EMGO Institute for Health and Care Research, VU University, Amsterdam, The Netherlands

**Keywords:** Guided self-help, Internet-based, Major depressive disorder, Randomised controlled trial, Negative effects of psychotherapy, Active control

## Abstract

**Background:**

Major depressive disorder (MDD) imposes a considerable disease burden on individuals and societies. A large number of randomised controlled trials (RCTs) have shown the efficacy of Internet-based guided self-help interventions in reducing symptoms of depression. However, study quality varies considerably. The aim of this study is to evaluate the efficacy of a new Internet-based guided self-help intervention (GET.ON Mood Enhancer) compared to online-based psychoeducation in an investigator-blinded RCT.

**Methods/design:**

A RCT will be conducted to compare the efficacy of GET.ON Mood Enhancer with an active control condition receiving online psychoeducation on depression (OPD). Both treatment groups will have full access to treatment as usual. Adults with MDD (*n* = 128) will be recruited and randomised to one of the two conditions. Primary outcome will be observer-rated depressive symptoms (HRSD-24) by independent assessors blind to treatment conditions. Secondary outcomes include changes in self-reported depressive symptom severity, anxiety and quality of life. Additionally, potential negative effects of the treatments will systematically be evaluated on several dimensions (for example, symptom deteriorations, attitudes toward seeking psychological help, relationships and stigmatisation). Assessments will take place at baseline, 6 and 12 weeks after randomisation.

**Discussion:**

This study evaluates a new Internet-based guided self-help intervention for depression using an active control condition (psychoeducation-control) and an independent, blinded outcome evaluation. This study will further enhance the evidence for Internet-based guided self-help interventions for MDD.

**Trial registration:**

German Clinical Trial Registration (DRKS): DRKS00005025

## Background

Major depressive disorder (MDD) is one of the most prevalent psychiatric disorders with a lifetime prevalence of more than 16% [1-3]. Moreover, MDD is related to a considerable quality of life decrement [4,5], increased mortality rates [6], and substantial economic costs [7-9]. Currently, MDD ranks as the fourth disorder with the highest disease burden and is projected to be the leading cause of disability in high-income countries by 2030 [10].

Although there is ample evidence for the effectiveness of psychotherapy in the treatment of depression [11,12], many individuals remain untreated [13]. People who could particularly benefit from treatment disregard treatment for several reasons including a lack of knowledge of what to do, prohibitive costs, anticipated negative (social) consequences or preference for self-help [14]. Moreover, those seeking help hardly receive immediate access to evidence-based treatment because of long waiting lists for psychotherapeutic treatment [14,15]. Limited availability of clinicians, geographical inaccessibility and difficulties to ‘attend therapy during usual business hours’ are further barriers.

Using the Internet to provide guided self-help interventions may help to overcome some of the limitations of traditional treatment services. Internet-based guided self-help strategies have several advantages over face-to-face approaches. These include: (1) interventions are more easily accessible at any time and place; (2) anonymity is assured when patients want to avoid stigmatisation; (3) a greater potential for the integration of acquired skills in daily life exists because of an emphasis on the participants’ active role in (guided) self-help interventions [16]; (4) participants can work at their own pace and go through materials as often as they want; (5) travel time and costs for both participants and clinicians are eliminated; (6) Internet-based interventions may attract people who do not (want to) make use of traditional mental health services [17]; and (7), Internet-based interventions are easily scalable, implying that only a small increase of therapeutic resources is required for reaching a greater proportion of the eligible population using these interventions.

Accumulating empirical evidence suggests that Internet-based interventions are well-accepted by participants [18] and effective in the treatment of MDD [19], subthreshold depression [20] and as maintenance treatment [21]. A systematic review of 19 randomised controlled trials (RCTs) evaluating Internet-based interventions for symptoms of depression in 2,996 individuals [19] found a mean effect size of d = 0.56. Interventions including at least some guidance by a clinician were more effective (d = 0.78) than interventions without guidance (d = 0.36). Cuijpers (2010) showed that guided self-help interventions for depression (and anxiety disorders) could have comparable effects to traditional face-to-face therapy even when they are directly compared to each other [22].

However, a recent methodological analysis of 75 RCTs of computer- and Internet-based interventions for psychiatric disorders [23] criticised the mean methodological quality of published trials in the field as rather low. They criticised, for example, that many studies used weak control conditions (that is, wait-list control), that most studies relied solely on participants’ self-report, and that they failed to include an independent assessment such as blind ratings or biological indicators. Moreover, they found that low overall methodological quality scores were associated with higher reported effect sizes. In fact, the latest systematic review in the field on Internet-based treatments for depression [19] did not include any study that used an independent outcome assessment by assessors blind to the treatment condition. Moreover, half of the included studies used a wait-list control-only comparison. To the best of our knowledge no study on Internet-based treatment for major depression published so far, has applied an independent, blinded outcome evaluation.

Another important issue not adequately addressed to date is the potential negative effects of Internet-based treatments for depression. One particularly unfavourable outcome of psychotherapy is deterioration of symptoms during treatment. However, RCTs evaluating psychological treatments seldom report the number of patients who had deteriorated while being treated [24]. Critics of Internet-based treatments have often emphasised that treatment might be provided that is less intensive than required to treat severely affected or symptomatic individuals [23]. This inadequate treatment allocation may result in less treatment expectations for psychotherapy in general and discourage individuals from seeking more intensive treatment. Hence, not only is there a pressing need for research on potential adverse events of psychotherapy in general [24] but also specifically for Internet-based treatments.

### Objective and research questions

The aim of this study is to evaluate the efficacy of a newly developed guided self-help Internet-based intervention (GET.ON Mood Enhancer) compared to an online psychoeducation on depression (OPD).

We expect that observer-rated depressive symptomatology assessed by raters blind to treatment conditions will be reduced to a greater extent in the intervention group than in the control condition. Moreover, we hypothesise that GET.ON Mood Enhancer is superior in terms of self-reported depressive symptoms, wellbeing, quality of life, symptoms of anxiety and problem-solving skills compared to the OPD-control condition. Potential negative effects with regard to (1) numbers of patients with symptom deteriorations, (2) attitudes towards seeking psychological help, and (3) other adverse events will be systematically evaluated. We hypothesise that both treatment conditions will not have a negative effect on attitudes towards seeking psychological help.

## Methods/design

### Design

**Figure 1:**
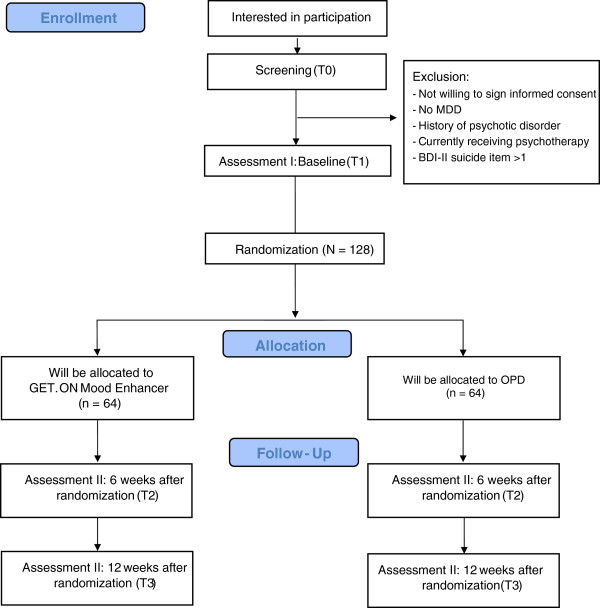
Study flow.

A two-arm RCT will be conducted to compare GET.ON Mood Enhancer with an online psychoeducation on depression (OPD) condition. Both treatment arms will have full access to treatment as usual. Assessments will take place at baseline (T1), post-treatment (6 weeks, T2) and 12-week follow-up (T3; see Figure 1 for a detailed overview of assessments). All procedures involved in the study will be consistent with the generally accepted standards of ethical practice approved by the University of Marburg ethics committee (No. 2013–08 K). The trial is registered in the German clinical trials register under DRKS00005025.

### Trial status

By the time of submission, recruitment for the trial was still ongoing. Recruitment started in June 2013 and will approximately last until February 2014.

### Participants and procedure

#### Inclusion and exclusion criteria

We will include adults (1) above the age of 18 years (2) with MDD according to DSM-5 criteria (3) who have Internet access, (4) have sufficient German skills in reading and writing (self-report), and (5) are willing to give informed consent. We will exclude subjects (1) with a history of manic/hypomanic episodes, (2) with a history of psychotic disorders, (3) currently receiving psychotherapy for any kind of mental health problems, (4) showing a notable suicidal risk as indicated by a score greater than 1 on BDI Item 9 (‘I feel I would be better off dead’), or (5) a change in antidepressive medication dosage/drug within 4 weeks before baseline.

#### Recruitment

Participants will be recruited via the GET.ON research website [25] that is announced in newspapers, on-air media and related websites. The research website provides information about the GET.ON Mood Enhancer training and details about another study evaluating GET.ON Mood Enhancer in a sample with subthreshold depression (Trial registration: DRKS00004709, [26]). Those participants who do fulfil criteria for a MDD are excluded from that study, and instead offered to participate in this trial.

#### Assessment of eligibility and randomisation

People who apply for study participation will receive an information letter with detailed information about the study procedures. Full written informed consent will be obtained from all participants. They will be informed that they can withdraw from the intervention and/or study at any time without any negative consequences. Nevertheless, we will ask those who withdraw from the trial treatment to attend all the remaining research appointments or at least provide minimal data (primary outcome measure). Applicants who continue to participate in the study will be asked to complete online screening questionnaires to assess the severity of their depressive symptoms (CES-D >16), whether they are currently receiving any kind of treatment for any mental health disease and whether they have a high suicidal risk (BDI Item 9 > 1). Subjects screened positive and are willing to give informed consent will be scheduled for a structured clinical interview (SCID) conducted via the telephone [27]. SCIDs will be conducted by trained clinicians. Participants meeting all of the inclusion and none of the exclusion criteria, who have completed the baseline assessment, and returned the informed consent form will enter the study and will be randomly allocated to study conditions. Randomisation will take place at an individual level. The allocation will be performed by an independent researcher not otherwise involved in the study using an automated computer-based random integer generator (randlist). The allocation will be concealed in advance from participants, researchers involved in recruitment, and therapists.

#### Assessments

Self-report and observer-rated assessments will take place at baseline, post-intervention and 12-week follow-up. See Figure 1 for a detailed overview. Self-report data will be collected using a secured online-based assessment system (AES, 256-bit encrypted), and observer-based assessments will be conducted via the telephone by trained interviewers. Observer-rated assessments will be recorded to examine inter-rater reliability. In case of disagreement, the two raters will discuss until a consensus is formed, and the agreed rating will be used for analysis. If no agreement is reached after discussion, the assessment will be rated by an experienced diagnostic-rater (gold standard), and this rating will be used for analysis.

#### Blinding

The research staff conducting the observer-based rating of depressive symptoms will be blinded to the condition the participants are assigned to. Considerable effort is undertaken to ensure blindness, including (1) an explanation to the participants why it is important not to inform the interviewer about the condition they were assigned to, (2) a written reminder in the interview manual for the interviewer to ask the participant not to mention anything about their randomisation status, (3) verbal reminders to the patient before the interview, and (4) a documentation after the assessment of whether or not the interviewer is still blind to the treatment condition. In the event of a blind violation, the interviewer will be changed to the following assessment. Participants know about the content of both conditions, but are blinded with regard to which intervention is the experimental and which the control condition.

### Intervention

#### GET.ON Mood Enhancer

GET.ON Mood Enhancer is a brief intervention consisting of six lessons with modules concerning psychoeducation, behavioral activation (BA), problem solving (PS) and relapse prevention. Additionally, participants are offered four modules that can be chosen based on individual need and/or preference. Additional modules are directed at sleep problems, relaxation techniques and dealing with worrying thoughts (see Table 1 for a session overview). Lessons consist of text, exercises and testimonials and also include interactive elements such as audio and video clips. A strong focus of the intervention lies on transfer tasks (homework assignments) to integrate newly acquired strategies and techniques into daily life. In the beginning of each subsequent lesson, participants are invited to reflect on their experiences with the newly acquired skills. The training is adaptive as the content is tailored to the specific needs of the individual participant by continuously asking participants to respond by choosing among various response options. Subsequent content is then tailored to the participant’s response. Using responsive web design, participants can follow the program on the Internet, a tablet or mobile phone. An integrated read-aloud function allows participants to follow narrated lessons. Participants are advised to complete at least one but preferably two lessons per week, because a previous meta-regression analysis about the role of treatment intensity for treatment outcome in the treatment of depression indicated that more frequent therapy sessions might be associated with a better outcome compared to a lower frequency of sessions [28]. Consequently, the training lasts about 3 to 6 weeks. We decided that we will not include more modules, because (1) a recent meta-analysis [19] found that web-based interventions for depression including more than seven modules were less effective (d = 0.36) than interventions with seven or less modules (d = 0 .75), and (2) we want to lower the threshold for individuals as much as possible. In terms of a stepped-care model participants will be encouraged to seek more intensive help in routine mental health services, if their progress is not sufficient at the end of the intervention. During the last treatment session, they will be provided with information about evidence-based treatments in routine mental healthcare and how to obtain access to it.

**Table 1:** Overview of GET.ON Mood Enhancer lessons

**Session**	**Topic**	**Content**
1	Psychoeducation	• Information about symptoms, causes and types of depression
		• Role of motivation
		• Introduction of the mood and activity diary
2	Behavioural activation (BA) I	• Relationship between activities and depression
		• Introduction of daily activity scheduling
3	Behavioural activation (BA) II	• Reflection on experiences with newly acquired skills and on activities/behaviour that might have influenced the mood (from now on in every session at the beginning)
		• Coping with difficulties with regard to daily activity scheduling
		• Goal setting for the upcoming days
		• Additional: sleeping problems
4	Problem-solving techniques (PST) I	• BA: reflection of goal attainment and goal setting for the upcoming days
		• PST: distinction between solvable and unsolvable problems
		• PST: introduction of 6-step PST procedure: (1) defining the problem, (2) defining the goal, (3) brainstorming about possible solutions and choosing the best one, (4) making a plan how to implement this solution, (5) putting the solution into practice, and (6) evaluating the outcome.
		• PST: choosing one personal problem, filling out steps 1 to 4, step 5 as homework
5	Problem-solving techniques (PST) II	• BA: reflection of goal attainment and goal setting for the upcoming days
		• PST: deepening the 6-step procedure: step 6 of the 6-step procedure: evaluating outcome; depending on the evaluation: coping with difficulties, revising steps 1 to 4 or choosing another problem
		• Additional: stop-worrying techniques
6	Relapse prevention	• BA: reflection goal attainment and goal setting for the future
		• PST: step 6 of the 6-step procedure: evaluating outcome
		• Evaluation: summarising gains and learned strategies
		• Developing an individual relapse prevention plan
		• Information about further healthcare services

The main modules used in GET.ON Mood Enhancer are based on evidence-based face-to-face manuals that have been found to be effective in the treatment of depression, such as behaviour therapy (BT) [29] and problem-solving therapy (PST) [30]. In BT, a strong focus rests on daily pleasurable activity scheduling that is integrated in each lesson. The PST elements implemented in GET.ON Mood Enhancer have been used in various web-based interventions, such as the Dutch web-based ‘Alles onder Controle’ course, which has been shown to be effective in reducing depressive symptomatology across several RCTs [31,32].

During the training, participants will be supported by an online therapist. Every participant will be assigned to one therapist for the duration of the study. The total time a therapist spends on a participant will be approximately 2 to 3 hours. Guidance is provided by psychotherapists in training supervised by an experienced clinician. Participants will communicate with their therapist through the internal messaging function of the system on which GET.ON Mood Enhancer is implemented. The guidance is mainly based on the supportive-accountability model of providing guidance in Internet-based interventions [33]. In the current study, the purpose of the guidance will be to support participants to adhere to the treatment modules, and therapists will not teach therapeutic techniques beyond techniques used in the treatment modules. All feedback from therapists is stored for supervision and adherence checks.

#### Psychoeducation on depression condition ***(*** OPD ***)***

The OPD intervention is also Internet-based and is implemented on the same platform as GET.ON Mood Enhancer. In the current study, the psychoeducational intervention is based on the patient version of the German S3-Guideline/National Disease Management Guideline Unipolar Depression [34]. It informs participants about the nature and evidence-based treatments of depression including information about symptoms, strategies to overcome depression and sources of help. In this study, the psychoeducational intervention requires no explicit homework assignments from the participants and provides no therapist support. Passive psychoeducational interventions have been shown to be effective in reducing depressive symptoms with a pooled standardised effect size of d = 0.26 [35].

### Treatment as usual

There will be no restriction on the use of treatment as usual in routine mental health services during the study period, such as psychotherapeutic and psychiatric treatment or antidepressant medication. However, to control for potential confounding effects, treatment utilisation and changes in the dosage of antidepressant medication intake will be monitored during the study period.

### Primary and secondary outcomes

The primary outcome will be observer-rated depression severity. In the secondary analyses, we will explore the effects of the treatments on self-reported depression severity, wellbeing, anxiety symptoms, quality of life and the number of patients who (1) responded and (2) are in remission. Moreover, we will also systematically evaluate potential negative treatment effects (that is, symptom deteriorations, negative effects on attitudes towards seeking psychological help; other adverse events).

### Measures

#### Primary outcome

The primary outcome will be observer-based rating of depressive symptom severity measured by the Hamilton Rating Scale for Depression HRSD_24_[36-38]. The HRSD is likely to be the most widely used clinician-rated scale for measuring depression in research. The self-report measure assesses depressed mood, vegetative and cognitive symptoms of depression, and anxiety symptoms. Items are rated on either a 5-point or a 3-point scale, and the total score is derived by summing the individual item scores. Higher scores indicate greater symptom severity. The HRSD is sensitive to change, inter-rater reliability is 0.90 [36], and the scale corresponds well with overall clinical ratings of severity [39,40]. The cutoff points of 10, 19, 27 and 35 represent the threshold for mild, moderate, severe and very severe depression, respectively.

#### Secondary outcomes

##### Depressive symptoms

As the secondary outcome, observer-based rating of depression will also be measured using the Quick Inventory of Depressive Symptomatology - Clinician-Rating QIDS-CR_16_[41,42]. The 16-item QIDS-CR_16_ is a brief clinician-report rating scale developed from the 30-item Inventory of Depressive Symptomatology [41-43]. In contrast to the HRSD, it evaluates only the nine depression criterion symptom domains (that is, sad mood, concentration, self-criticism, suicidal ideation, interest/involvement, energy/fatigability, sleep disturbance, appetite/weight change and psychomotor agitation/retardation) from the Diagnostic and Statistical Manual of Mental Disorders [44] during the prior 7 days. Each item is scored on a scale from 0 to 3 points, with higher scores indicating higher symptom severity. This measure has shown good psychometric properties, such as strong internal consistency (α = 0.85), concurrent validity and sensitivity to symptom change in patients with MDD [42]. The cutoff points of 6, 11, 16 and 21 represent the threshold for mild, moderate, severe and very severe depression, respectively.

Self-reported depressive symptoms will be assessed with the Patient Health Questionnaire (PHQ-9). This measure has comparable sensitivity and specificity to many other depression measures, although it is only half the length. Its internal reliability reaches values between α = 0.86 and 0.89 [45,46]. Each item assesses the frequency of a symptom in the last 2 weeks on a scale ranging from 0 (‘not at all’) to 3 (‘nearly every day’). In contrast to the primary outcome measure HRSD_24_, the PHQ-9 only assesses frequency of symptoms, not their intensity. The total score ranges from 0 to 27, with a higher score indicating more frequent symptoms. The cutoff points of 5, 10, 15 and 20 represent the threshold for mild, moderate, moderately severe and severe depression, respectively [45,46].

##### Quality of life

Health-related quality of life will be assessed with the SF-12v1 Health Survey [47]. The SF-12v1 has 12 items covering eight health domains (physical functioning, physical and emotional role functioning, body pain, general health, vitality, social functioning and mental health). The SF-12 generates a physical and a mental health summary score.

##### Anxiety

Anxiety will be measured with the anxiety subscale of the Hospital Anxiety and Depression Scale HADS-A [48]. The anxiety subscale consists of seven questions and each is scored from 0 to 3 with total scores ranging from 0 to 21. A score between 0 and 7 indicates no anxiety, between 8 and 10 possible anxiety, and above 11 or 12 a clinical anxiety disorder. Psychometric properties are well established [49].

##### Problem-solving skills

Problem-solving ability (that is, generalised appraisal, beliefs, expectancies and emotional responses) will be measured with two subscales of the Social Problem-Solving Inventory-Revised (SPSI-R). The positive problem orientation (PPO) subscale represents a constructive dimension, and the negative problem orientation (NPO) subscale is viewed as a dysfunctional dimension. Cronbach’s alphas are α = 0.76 for the PPO dimension and 0.83 for the NPO dimension [50].

##### Behavioural activation

Participants’ activation towards goals or values and pleasant activities and avoidance behaviours will be measured with the BADS-Short Form BADS-SF [51]. The BADS-SF entails nine items comprising two subscales (activation and avoidance). The items are rated on a 7-point Likert-type scale. Higher scores indicate that the participant scores high on the area of interest. The internal consistency is α = 0.82 [51].

##### Wellbeing

Psychological wellbeing will be measured by the World Health Organization’s (WHO) 5 Wellbeing Index [52]. Participants indicate for each of the five statements, which one is closest to how they have been feeling over the prior 2 weeks. Each question is scored from 0 to 5 with the total score ranging from 0 to 25. Higher scores indicate better wellbeing.

##### Problematic alcohol use

The Alcohol Use Disorders Identification Test (AUDIT) is a 10-item questionnaire designed by the WHO to screen for hazardous alcohol intake. It assess three conceptual domains: alcohol intake (items 1 to 3), dependence (items 4 to 6) and adverse consequences (items 7 to 10). Sum-scores can range from 0 to 40, and the generally accepted cutoff point for identifying a potential alcohol problem is 8 [53].

##### Treatment credibility/patient expectancy

Training credibility and participants’ expectancy for improvement will be measured with the credibility and expectation questionnaire (CEQ). The CEQ consists of six items which are rated on a 9- or sometimes 10-point Likert scale. The psychometric properties of the instrument are well established [54].

##### Course evaluation

In absence of a standardised measure for evaluating course satisfaction in Internet-based treatments, user satisfaction will be measured with a self-designed questionnaire based on the Client Satisfaction Questionnaire CSQ-8, German Version [55,56]. This self-report measure consists of eight items measuring the global client satisfaction with the Internet-based training. Previous research indicated a high internal consistency [57].

##### Response and remission

Participants will be coded as responders when they demonstrate a reliable change according to the widely used reliable change index (RCI) [58]. Participants with an RCI of 1.96 or above on the primary outcome measure HRSD_24_ will be considered responders. Remission is defined a priori as a non-pathological score of ≤10 on the HRSD_24_.

##### Negative effects

Negative effects will be measured on (1) symptom deterioration during treatment (2), attitudes towards seeking psychological help, and (3) other adverse events.

(1) *Symptom deteriorations*

Patients will be classified as deteriorated when they display a reliable negative change (−1.96) in the primary outcome measure HRSD_24_ according to the reliable change index, proposed by Jacobsen & Truax (1991) [58].

(2) *Negative effects on attitudes towards seeking professional psychological help*

A potential negative influence on attitudes towards seeking mental healthcare service utilisation will be measured with the Attitudes Toward Seeking Professional Psychological Help Scale-SF ATSPPH-SF [59]. The ATSPPH-SF consists of 10 items that are rated on a 4-point Likert scale from 0 to 3, yielding a total score ranging from 0 to 30. High scores indicate more positive treatment attitudes. The instrument showed good psychometric properties [60].

##### Other adverse events

Other adverse events will be measured with the negative effects of psychotherapy inventory (INEP). The INEP is a relatively new measure and was developed for assessing systematic and potentially negative effects of psychotherapeutic interventions in different domains. The version used in this study consists of 15 items assessing any negative effects participants experienced within or after the completion of the Internet-based training on (1) negative intrapersonal changes (for example, ‘During treatment or since the end of my therapy, I suffered from suicidal thoughts or intentions for the first time ever’), (2) negative effects in an intimate relationship (for example, ‘My partner is or has been jealous of my therapist’), (3) family/friends (for example, ‘The relationships with my friends has worsened’), (4) perceived dependence on the psychotherapist/psychotherapeutic intervention (for example, ‘I feel dependent on my therapist’), and (5) stigmatisation (for example, ‘I am anxious that my colleagues or friends could find out about my psychotherapy’). The items are rated on a 4-point Likert scale (0 = no agreement at all; 3 = total agreement). Chronbach’s alpha is α = 0.85 (Table 2).

**Table 2:** Overview of measurements

		**Time of measurement**		
**Instrument**	**Aim**	**T1 (Baseline)**	**T2 (6 weeks)**	**T3 (12 weeks)**
**SCID**	Diagnostic interview	x		
**HRSD**_**24**_	Hamilton Rating Scale for Depression	x	x	x
**QIDS**-**C**_**16**_	Quick Inventory for Depressive Symptomatology	x	x	x
**PHQ**-**9**	Depressive symptomatology	x	x	x
**WHO**-**5**	Wellbeing	x	x	x
**SF**-**12**	Quality of life	x	x	x
**HADS**-**A**	Anxiety symptoms	x	x	x
**SPSI**-**R**	Problem-solving skills	x	x	x
**BADS**-**SF**	Behavioural activation	x	x	x
**CEQ**	Patient expectancy/treatment credibility	x		
**ATSPPH**-**SF**	Attitudes toward seeking psychological help	x	x	
**BFI**	Big Five Inventory	x		
**INEP**	Inventory of negative effects in psychotherapy		x	x
**CSQ**-**8**	Clients’ satisfaction with the online training		x	
**AUDIT**	Alcohol use disorder identification test	x		
**Other questions**	Socio-demographics	x		

### Sample size calculation

We aim to include 128 participants. This sample will allow us to detect a between-group effect size (ES) of d = 0.50 with a power (1-ß) of 80% and an alpha of 0.05 (calculated using PASS 12). The most recent meta-analytic review found a mean ES of d = 0.78 for therapist-supported web-based interventions for depression [19]. However, most of the included studies used a waiting-list control comparison only, and these studies showed a considerably larger ES than those studies including a treatment-as-usual condition. Our comparison condition will include psychoeducation and full access to treatment as usual and participants are encouraged to seek further help. Thus, we expect a somewhat smaller ES of d = 0.50 at post-treatment.

### Statistical analyses

The clinical trial will be conducted in compliance with the Declaration of Helsinki, the CONSORT guidelines, GCP and the protocol. Aiming at an intention-to-treat design [61] we will include all participants who will be randomly assigned to one of the two conditions. Additional per protocol analyses (PPA) will be conducted, including only participants’ satisfying protocol treatment (completed at least 4 of 6 modules). Mixed-model analyses of variance will be conducted to explore the effects of the treatments on all continuous outcomes. Missing data will be handled using multiple imputations (MI). MI is especially robust with respect to missing data [62]. Nevertheless, to assess systematic effects of non-ignorable missing data, pattern mixture analyses for multi-level longitudinal approaches [63] will be conducted. To determine if the treatment effect is dependent on missing data, the missing-data pattern of each participant will be first coded and then included in a three-way interaction (missing pattern × condition × change in depression severity) in the main outcome analyses. If no significant interactions between missing-data pattern and treatment outcome are found, we will conclude that no missing data bias occurred in the results. For all mixed-model analyses, Cohen’s d [64] will be calculated by standardising the differences between baseline and follow-up scores by the pooled standard deviation of the baseline scores. Response and remission rates will be compared across groups with the help of contingency tables and χ2 tests. We will also calculate the number needed-to-be-treated (NNT) with GET.ON Mood Enhancer to achieve one response and remission, respectively, compared to the control group. We will also calculate the number needed-to-harm, which indicates the number of participants treated in the experimental condition for one extra person to have a symptom deterioration. Because the differential risk of the intervention ideally needs to be set into relation with its benefits we will also calculate a benefit-risk ratio [65]. Benefit-risk ratio will be calculated by dividing the NNH to achieve one symptom deterioration through the NNT to achieve one response. If this benefit-risk ratio is greater than 1, the benefits outweigh the risks; if it is 1, the balance between benefit and risks are equal across the groups; if it is less than 1, the risks outweigh the benefits (LIT). Benefit-risk ratios will only be calculated when differences in risk for symptom deteriorations are statistically significant.

For other negative effects, we will use independent t-tests for continuous, χ^*2*^ for categorical, and logistic regression for binary outcomes. In a pre-planned subgroup analysis, we will explore negative effects of GET.ON Mood Enhancer on attitudes towards seeking psychological help in patients who do not reach the response criteria up to post-assessment (6 weeks). For all statistical analyses, significance level will be set at *P* <0.05. All analyses will be conducted using SPSS 20.

## Discussion

MDD is a highly prevalent disorder associated with a considerable loss in quality of life, increased mortality rates and substantial economic costs. Several reasons lead to the fact that patients remain untreated although they are in need of help. Internet-based guided self-help approaches could be an attractive, efficient and cost-effective approach to offer an evidence-based treatment alternative. Numerous studies have shown the acceptance and efficacy of guided self-help interventions in the treatment of depression. However, recent reviews have criticised that the methodological quality of studies are low and more high quality studies are needed, before such Internet-based therapies are widely disseminated. Moreover, as is also the case for face-to-face psychotherapy, hardly anything is empirically known about the potential negative effects of Internet-based guided self-help approaches. This study will evaluate a new Internet-based intervention for depression (GET.ON Mood Enhancer) compared to an online psychoeducation control in an investigator-blinded RCT. Special emphasis will be given to systematically evaluate potential negative effects, such as symptom deterioration, effects on attitudes towards seeking psychological help and other adverse events (that is, perceived intrapersonal negative change, relationships, friendships, family, therapeutic malpractice and stigmatisation).

This study will also have some limitations. First, as in most longitudinal studies we will need to deal with the problem of missing values. Although the planned adjustment for missing data (Multiple Imputation) is a highly recommended method to handle missing data [62], we will nevertheless additionally conduct pattern-mixture analyses [63] to minimise a possible risk for bias. Second, ideally the risk for negative effects when providing an Internet-based guided self-help intervention to patients with MDD needs to be compared with the risk for negative effects in face-to-face psychotherapy. However, a systematic evaluation of potentially negative effects of treatment has seldom been conducted for face-to-face psychotherapeutic interventions for Major Depression. Therefore, drawing conclusions on the differential risk of this guided self-help treatment compared to traditional psychotherapeutic treatment will not be possible. Third, the comparison condition does not control for unspecific effects through human support. Finally, the study sample will be too small to test for potential moderating effects. Thus, which patients are likely to profit from this type of treatment delivery or which patients are especially likely to experience negative effects will remain unclear.

There will also be several strengths of this study, including the strong methodology of a randomised controlled design with an active control condition, outcome assessment with validated assessments by independent raters blind to treatment condition, a sample defined by a standard diagnostic measure, an appropriate statistical analyses plan and handling of missing data with state of the art methods. Given these strengths, the results of the study should further enhance the evidence-base for Internet-based guided self-help interventions for Major Depression. Moreover, a systematic evaluation of potentially negative effects of an Internet-based treatment as it is conducted in the present study has to the best of our knowledge not been conducted before. This study will therefore provide valuable information to the field as the basis for a wide dissemination of such concepts.

Overall, to overcome the gap between the need for treatment and evidence-based treatment availability and treatment utilisation, (cost-) effective low-threshold interventions are needed that are accessible for as many people as possible. Internet-based guided self-help interventions might be a promising strategy not only as a first step in a stepped-care approach but by providing treatments that are more intensive when patients fail to respond. If at some point this strategy becomes classified as evidence-based treatment, such approaches could provide evidence-based services to patients in areas or countries where psychotherapeutic treatment is not readily available [66]. If the proposed trial shows that the investigated intervention is not only effective in reducing depressive symptoms but also shows an acceptable risk-benefit balance, it would further strengthen the arguments for a wide dissemination of Internet-based guided self-help approaches for Major Depression.

## Competing interests

MB is a minority shareholder of Minddistrict GmbH, which will provide the technical platform for the web-based interventions.

## Authors’ contribution

MB obtained funding for this study. DE, CB, JR, LB and DL contributed to the development of the GET.ON Mood Enhancer training. DE was responsible for the initial draft of design of the study, LB, PC, HR, HB, MB and DL further contributed to the study design. DE drafted the manuscript. LB, JR and CB are responsible for trial management and carrying out the diagnostic assessments. All authors contributed to the further writing of the manuscript. All authors read and approved the final manuscript.
